# Development of a Test Card Based on Colloidal Gold Immunochromatographic Strips for Rapid Detection of Antibodies against Theileria equi and Babesia caballi

**DOI:** 10.1128/spectrum.02411-21

**Published:** 2022-02-23

**Authors:** Guangpu Yang, Kewei Chen, Wei Guo, Zhe Hu, Ting Qi, Diqiu Liu, Yaoxin Wang, Cheng Du, Xiaojun Wang

**Affiliations:** a State Key Laboratory of Veterinary Biotechnology, Harbin Veterinary Research Institute, Chinese Academy of Agricultural Sciences, Harbin, China; Weill Cornell Medicine

**Keywords:** *Babesia caballi*, equine, piroplasmosis, *Theileria equi*, colloidal gold immunochromatographic test

## Abstract

Equine piroplasmosis (EP) is a serious problem in the horse industry, and controlling EP is critical for international horse trading. EP is caused by two apicomplexan protozoan parasites, Theileria equi and Babesia caballi. Rapid and accurate methods that are suitable for detecting these parasites in the field are crucial to control the infection and spread of EP. In this study, we developed a card to detect antibodies against T. equi and B. caballi based on two colloidal gold immunochromatographic strips according to the principle of the double-antigen sandwich. The proteins equi merozoite antigen 1 (EMA1) and rhoptry protein BC48 are commonly used as diagnostic antigens against T. equi and B. caballi, respectively. On the strip, the purified EMA1 or BC48 protein labeled with colloidal gold was used as the detector, and nitrocellulose membranes were coated with EMA1 or BC48 and the corresponding MAb as the test and control lines, respectively. The protocol takes 10 to 15 min and requires no specialized equipment or chemical reagents, and one test can detect two EP pathogens in one card. Specificity tests confirmed there was no cross-reactivity with sera positive for common equine pathogens. Using a commercial competitive enzyme-linked immunosorbent assay (cELISA) kit for comparison, 476 clinical samples were tested with the card. The coincidence rates were 96.43% and 97.90% for T. equi and B. caballi, respectively. The field trial feedback was uniformly positive, suggesting that this diagnostic tool may be useful for controlling the spread of T. equi and B. caballi.

**IMPORTANCE** Equine piroplasmosis (EP), caused by Theileria equi and Babesia caballi, is an important tick-borne disease of equines that is prevalent in most parts of the world. EP is considered a reportable disease by the World Organization for Animal Health (OIE). The accurate diagnosis and differentiation of T. equi and B. caballi are very important for the prevention, control, and treatment of EP. Therefore, we developed a double-antigen sandwich colloidal gold immunochromatography assay (GICG) to detect T. equi and B. caballi. Two GICG strips were assembled side by side on one card for the detection of T. equi and B. caballi, and the two EP pathogens could be detected in one test. This method was simple, rapid, and specific for the detection of EP; therefore, compared to the previous methods, this method is more suitable for pathogen diagnosis in the field.

## INTRODUCTION

Equine piroplasmosis (EP), which is caused by Theileria equi and Babesia caballi, has emerged as an important protozoan infection (1). These parasites are widespread in tropical, subtropical, and certain temperate regions where tick vectors are present (2). The socioeconomic impact of the disease and the restrictions in trading infected animals led the World Organization for Animal Health (OIE) Animal Health Code to categorize EP as a notifiable disease (3). The occurrence of disease causes setbacks in the international movement of horses; for this reason, many countries have introduced stringent animal movement restrictions to prevent the introduction of T. equi and B. caballi into disease-free areas (4, 5). This highlights the need for robust and effective measures of control against the disease in countries of endemicity and for combined high-sensitivity serological studies to enhance the detection of animals exposed to the parasites that cause EP.

The current techniques for diagnosing T. equi and B. caballi infections include microscopic examination, the complement fixation technique (CFT), the indirect fluorescence antibody test (IFAT), competitive inhibition enzyme-linked immunosorbent assay (cELISA), and PCR (6–9). Light microscopy examination of thin blood smears is difficult in the case of acute and early infections, and discernible mixed-infection samples are rare. Several methods, including CFT, IFAT, and cELISA, are available for the detection of parasite-specific antibodies in infected horse sera (10). Currently, OIE considers cELISA to be the preferred test for EP in the international horse trade (3). However, these methods are tedious or require expensive equipment and highly skilled personnel, and thus, these methods are better suited for the laboratory than in the field. The sensitivity of PCR methods for detecting T. equi and B. caballi has been shown to be higher than that of traditional diagnosis methods (11–15), but these methods are still relatively time-consuming and require complex procedures. Although it may be difficult to clinically differentiate T. equi and B. caballi, their differentiation may be important for successful treatment (16, 17). The accurate diagnosis of T. equi or B. caballi infection is conducive to the implementation of treatment and prevention measures. One-step immunochromatography using gold nanoparticles has frequently been used to detect parasite antibodies and parasite antigens. Hence, in this work, we designed and developed a test card containing two colloidal gold immunochromatographic (GICG) strips for the detection of T. equi and B. caballi antibodies in equine serum. The GICG strip employs a double-antigen-sandwich immunoassay format, in which the expressed recombinant protein is applied to capture target antibodies at the test (T) line of the strip. The reaction of gold-labeled antigens with the corresponding antibodies can result in a visible color reaction (18–20). The distinctive advantages of colloidal gold particles are that they can be directly observed without staining and have a high resolution that provides accurate positioning of results. GICG assays have been increasingly applied to more research fields because they provide rapid analysis, have high sensitivity and low cost, and are simple to operate (21–24).

The erythrocytic-stage surface protein equi merozoite antigen 1 (EMA1) is an important antigen that induces specific neutralizing antibody responses in infected animals and, thus, can potentially be used in the diagnostic assay for T. equi (25, 26). BC48 is a rhoptry protein of the merozoites of B. caballi and has a molecular mass of 48 kDa, and BC48 was previously evaluated as a promising antigen for the serological detection of antibodies to B. caballi (27, 28).

**FIG 1:**
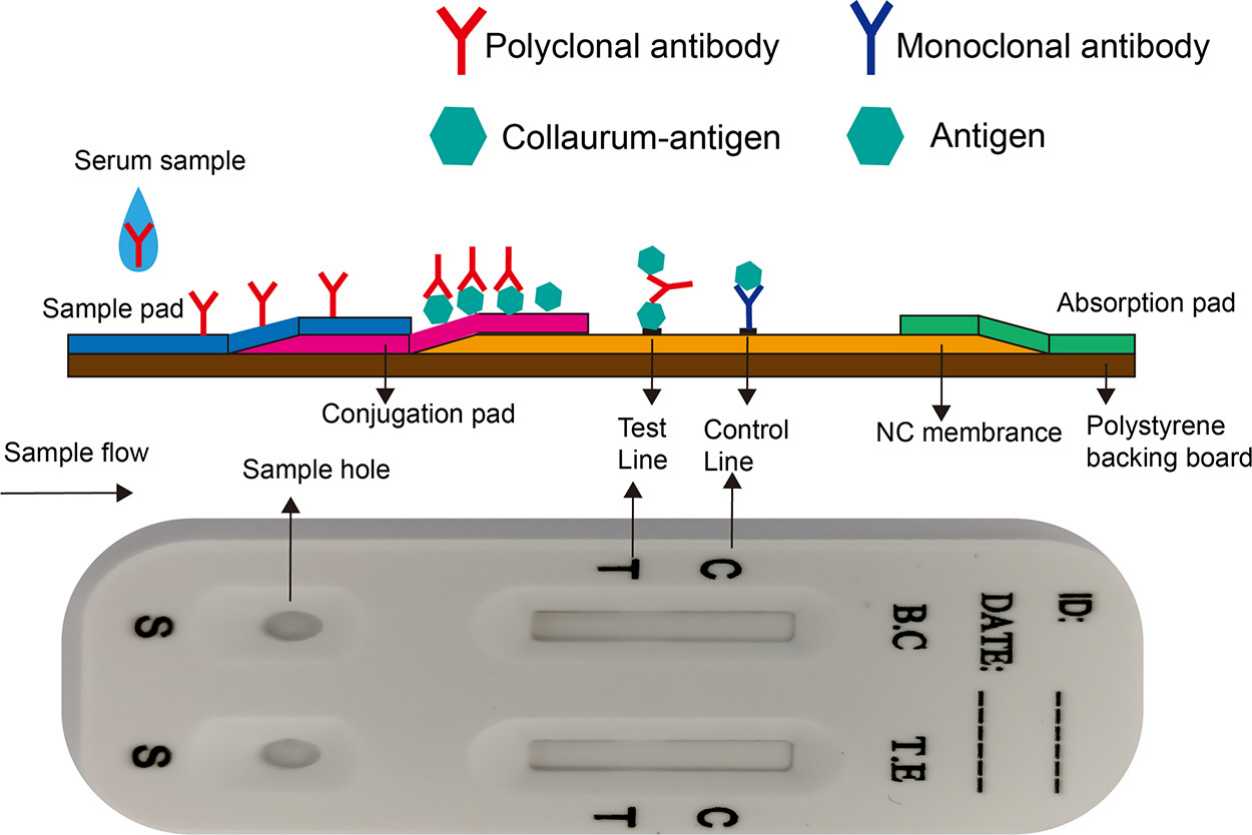
Schematic diagram of the colloidal gold test strip and the final product of the colloidal gold test card package. The test card has two loading holes and two result display areas, corresponding to the colloidal gold strips for T. equi (T.E) and B. caballi (B.C), respectively. Every test strip included 3 pads (sample pad, conjugate pad, and absorbent pad), a nitrocellulose membrane, and a polystyrene backing board. The conjugate pad contained gold-labeled EMA1 or BC48, which provided green and yellow colors. There were 2 lines on the nitrocellulose membrane: the test line (T line) and the control line (C line). The T line contained gold-labeled EMA1 or BC48, and the C line contained MAb against EMA1 or BC48.

**FIG 2:**
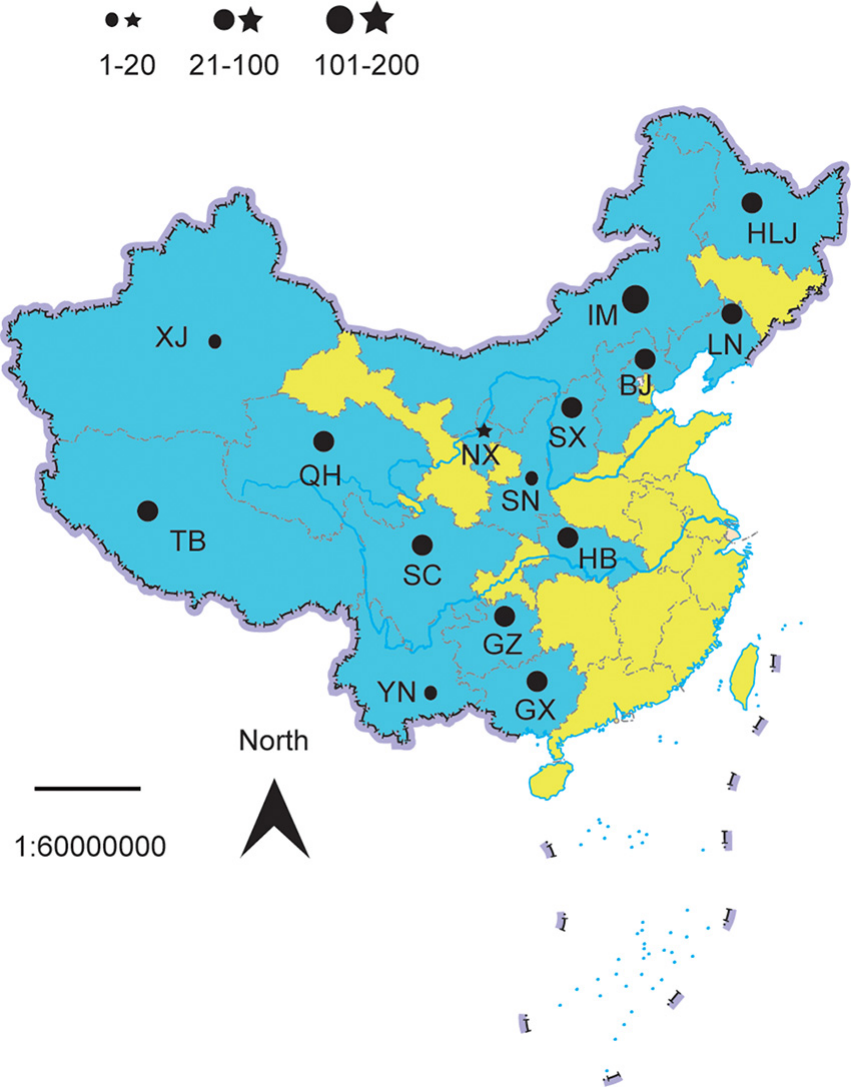
Map of China showing the study areas where serum samples were collected from horses and donkeys. Blue color represents the provinces where samples were collected and yellow color represents the provinces where samples were not collected. Black solid circles and stars represent the different locations from which serum samples from horses and donkeys, respectively, were taken. The sizes of the black solid circles and stars represent the amount of sampling performed. The names of provinces and regions are abbreviated as follows: BJ, Beijing; GX, Guangxi; GZ, Guizhou; HLJ, Heilongjiang; HB, Hubei; IM, Inner Mongolia; LN, Liaoning; NX, Ningxia; QH, Qinghai; SX, Shanxi; SN, Shannxi; SC, Sichuan; TB, Tibet; XJ, Xinjiang; YN, Yunnan. The standard map was downloaded from the Ministry of Natural Resources of the People’s Republic of China (http://bzdt.ch.mnr.gov.cn/browse.html?picId=%224o28b0625501ad13015501ad2bfc0047%22).

In this study, two GICG strips that could detect antibodies of T. equi and B. caballi were developed by using the recombinant proteins EMA1 and BC48 and the corresponding monoclonal antibodies (MAbs). We assembled these two GICG strips in one small plastic card slot to form one test card (Fig. 1). Therefore, with this card, one test could detect and distinguish the antibodies of two pathogens. Furthermore, the sensitivity and specificity of this card were routinely evaluated. In addition, 476 serum samples from 15 provinces of China (Fig. 2), including Beijing, Guangxi, Guizhou, Heilongjiang, Hubei, Inner Mongolia, Liaoning, Ningxia, Qinghai, Shanxi, Shannxi, Sichuan, Tibet, Xinjiang, and Yunnan, were tested and the results compared with the results of a commercial cELISA kit, and the positivity rates of serum samples in the sampling areas were obtained. This method was simple, rapid, and specific for the detection of EP; therefore, compared to the previous methods, this method is more suitable for pathogen diagnosis in the field.

## RESULTS

### Expression and purification of recombinant proteins EMA1 and BC48.

**FIG 3:**
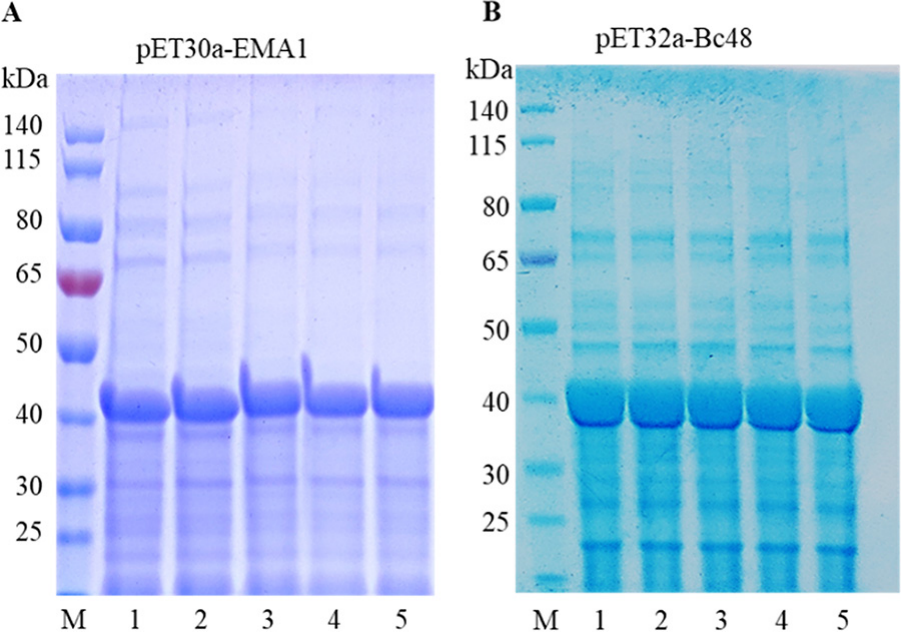
Purification and identification of recombinant EMA1 and BC48. (A) SDS-PAGE analysis of purified recombinant EMA1 using a 12% acrylamide gel. Lanes: M, protein markers; 1 to 5, purified protein of pET-30a-EMA1. (B) SDS-PAGE analysis of purified recombinant BC48 using a 12% acrylamide gel. Lanes: M, protein markers; 1 to 5, purified protein of pET-32a-BC48.

The constructed prokaryotic expression plasmids pET-30a-EMA1 and pET-32a-BC48 were transformed into BL21(DE3) competent Escherichia coli cells for induction. The highest soluble expression was achieved at an induction temperature of 25°C, an IPTG (isopropyl-β-d-1-thiogalactopyranoside) concentration of 0.5 mM, and an induction time of 8 h. Protein purification was performed at 25°C, and the SDS-PAGE verification results are shown in Fig. 3A and B. The results showed that the molecular weight of the EMA1 protein was approximately 42 kDa and that the BC48 protein was approximately 40 kDa.

### Production of the MAbs against EMA1 and BC48.

**FIG 4:**
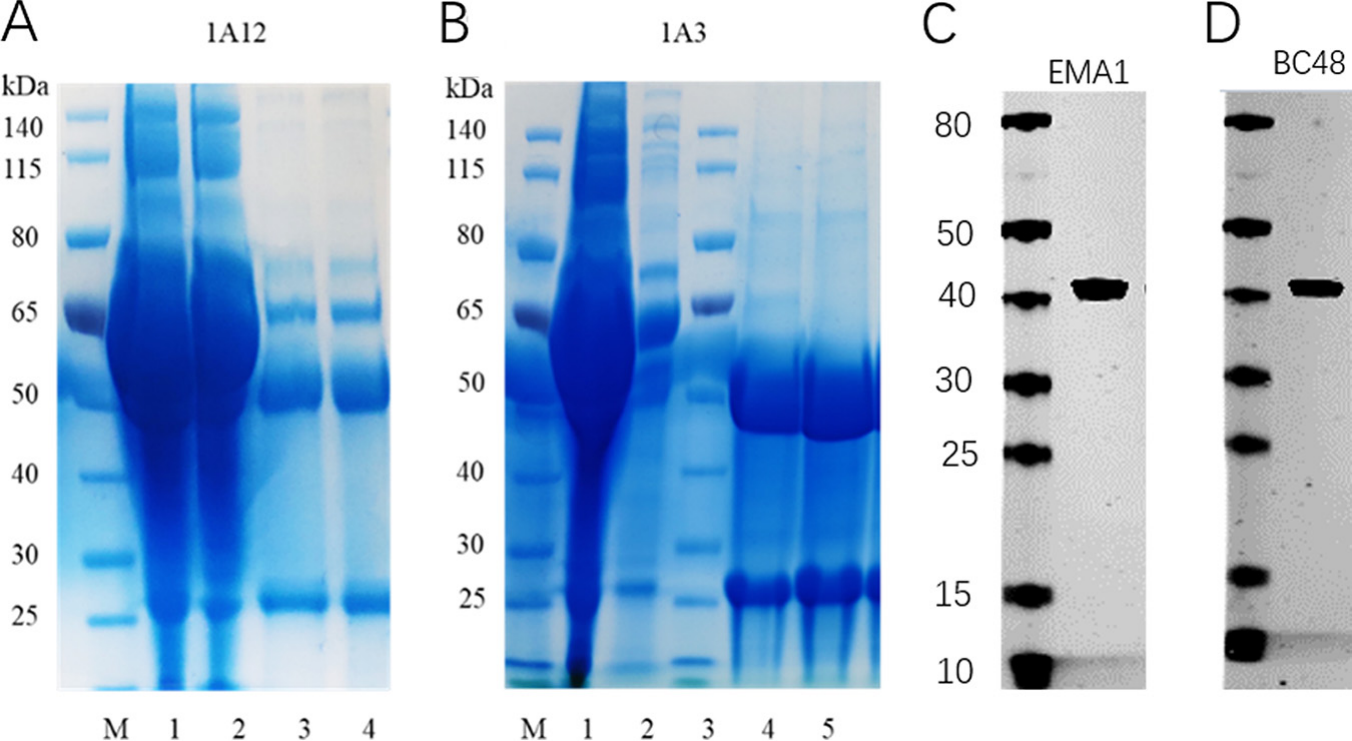
SDS-PAGE and Western blot analysis of purified monoclonal antibodies. (A) Purification of EMA1 MAb 1A12. Lanes: M, protein markers; 1, flowthrough; 2, wash 1; 3, elution 1; 4, elution 2. (B) Purification of BC48 MAb 1A3. Lanes: M and 3, protein markers; 1, flowthrough; 2, wash 1; 4, elution 1; 5, elution 2. (C) Western blot detection of EMA1 by MAb 1A12. (D) Western blot detection of BC48 by MAb-1A3.

Serum samples were collected from mice immune to EMA1 or BC48 1 week after the third immunization, and the optical density at 450 nm (OD_450_) was detected by indirect ELISA. SP2/0 cells were mixed with spleen cells at a ratio of 1:8 and centrifuged. They were fused with polyethylene glycol 1450 (PEG 1450) (Sigma, USA) at 37°C and washed twice with RPMI 1640 medium (Sigma, USA). After homogenized dilution, the supernatant was added to a 96-well plate lined with cultured cells. After three rounds of hybridoma cell screening, hybridoma cells that could secrete MAbs against 1A12 (EMA1) or 1A3 (BC48) were selected. After identification by using an antibody subtype kit (Southern Biotech, USA), MAbs 1A12 (EMA1) and 1A3 (BC48) were both determined to be IgG1 subtypes. The cell line that secreted 1A12 or 1A3 was expanded and then injected into the abdominal cavity of mice to prepare ascites and purified antibodies, as shown in Fig. 4A and B.

The reactivity of 1A12 or 1A3 was tested via Western blot analysis with recombinant EMA1 or BC48, and the results showed that MAbs 1A12 and 1A3 clearly reacted with the recombinant EMA1 and BC48 proteins, respectively (Fig. 4C and D).

### Preparation of colloidal gold conjugate.

In the GICG strip system, the purified protein EMA1 or BC48 and the purified MAb 1A12 or 1A3 were sprayed linearly on a nitrocellulose (NC) membrane (Millipore, USA) as the test and control lines, respectively. The nitrocellulose membranes sprayed with purified EMA1 or BC48 protein and purified 1A12 or 1A3 MAb, as well as the conjugate pad, sample pad, and absorbent pad, were cut into 4-mm-wide strips and assembled on an adhesive card. A test of the strip was performed with T. equi- and B. caballi-positive and -negative serum. As shown on the strips, a positive reaction was indicated by purplish-red on both the test and control lines. In contrast, a negative reaction resulted in the test line having no color. The prepared GICG strips for T. equi and B. caballi were installed in two specific card slots to form a T. equi*-*B. caballi combined-detection card, as shown in Fig. 1. In this way, the test strips for the T. equi and B. caballi detection antibodies could be installed on one card, making field testing more convenient.

### Specificity and sensitivity of the test card.

**FIG 5:**
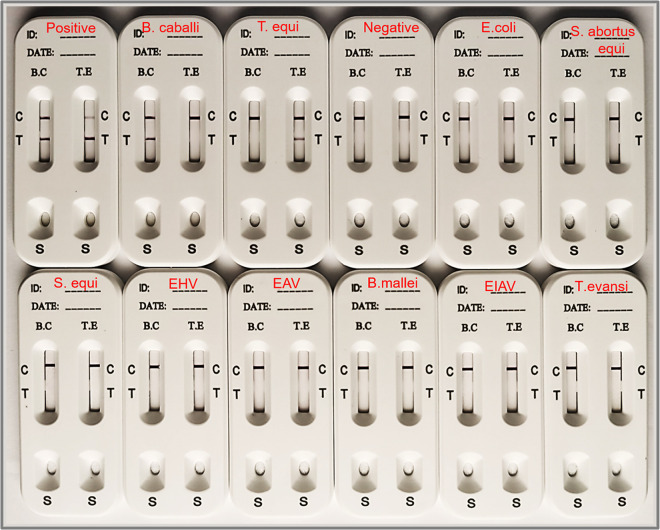
Specificity of the strip relative to the results of the cELISA. In the top row, the test strips from left to right characterize the detection results for T. equi (T.E)- and B. caballi (B.C)-mixed-positive serum, B. caballi-positive serum, T. equi-positive serum, both B. caballi- and T. equi-negative serum, E. coli-positive serum, and Salmonella *abortus equi*-positive serum. In the bottom row, the test strips from left to right characterize the detection results for S. equi-positive serum, EHV-4-positive serum, EAV-positive serum, B. mallei*-*positive serum, EIAV-positive serum, and T. evansi*-*positive serum.

**FIG 6:**
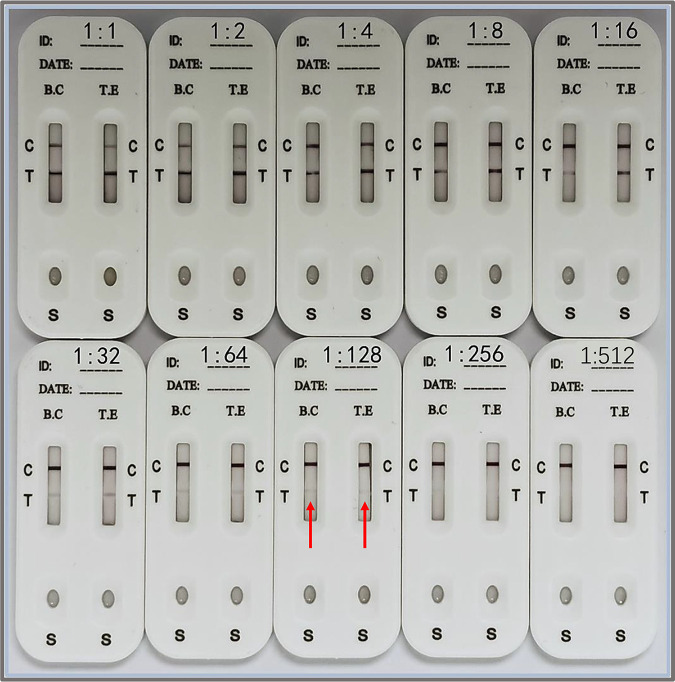
Sensitivity of the strip relative to the results of the cELISA. The sensitivity of the immunochromatographic test strip was evaluated by serial dilutions of separate serum samples positive for T. equi (T.E) or B. caballi (B.C). In the top row, the test strips from left to right characterize the detection results for 1:1, 1:2, 1:4, 1:8, and 1:16 dilutions. In the bottom row, the test strips from left to right characterize the detection results for 1:32, 1:64, 1:128, 1:256, and 1:512 dilutions.

To verify the specificity of this detection method, we tested sera positive for E. coli, Salmonella *abortus equi*, Streptococcus equi, equine herpesvirus type 4 (EHV-4), equine arteritis virus (EAV), Burkholderia mallei, equine infectious anemia virus (EIAV), and Trypanosoma evansi, but no band was detected at the test line (T line), indicating that the result was negative (Fig. 5). The results showed that there was no cross-reaction between sera positive for the above-named pathogens and T. equi and B. caballi. At the same time, serum positive for T. equi was tested, and there was no band at the T line of the B. caballi detection strip. When serum positive for B. caballi was tested, there was no band at the T line of the T. equi detection strip. It was demonstrated that there was no cross-reaction between T. equi and B. caballi. The above-described results showed that our T. equi*-*B. caballi combined detection card had good specificity. In the sensitivity test, the sensitivity was tested with separate serum samples, a weak band could be detected at the T line when serum positive for T. equi or B. caballi was diluted at a 1:128 ratio (Fig. 6).

### Comparison of the test card with a commercial ELISA.

**FIG 7:**
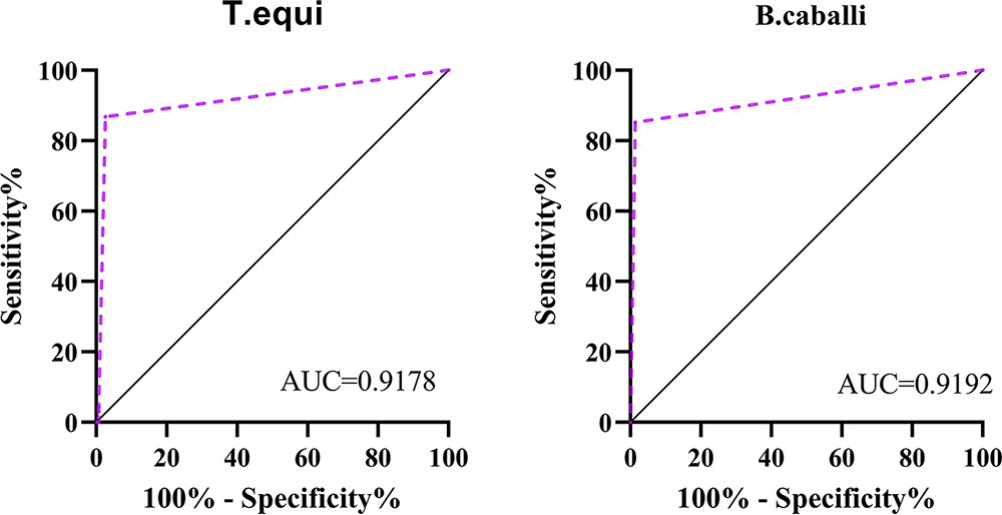
ROC curve analysis of the test card assay for clinical sample testing. ROC curves were plotted by calculating the sensitivity and specificity of the test card results relative to the results of the cELISA.

The established T. equi*-*B. caballi combined-detection card was applied to test 476 clinical serum samples from horses and donkeys in 15 provinces of China (Fig. 2), and the results were compared with those of a commercial cELISA kit (VMRD, USA) for the detection of antibodies against T. equi and B. caballi. Compared with the cELISA method, the areas under the receiver operating characteristic (ROC) curves (AUCs) of the GICG assay were 0.9178 for T. equi and 0.9192 for B. caballi. Both values were statistically significant (*P* < 0.001) compared with the value for significance of 0.05 (Fig. 7).

As shown by the results in Table 1, the positive rate of T. equi detected by our test card was 13.45% (64/476), and the positive rate of T. equi detected by the cELISA kit was 13.66% (65/476) (Table 1). The coincidence rate of T. equi between the test card and cELISA was 96.43% (459/476) (Table 2). The positive rate of B. caballi detected by our test card was 6.09% (29/476), and the positive rate of B. caballi detected by the cELISA kit was 5.67% (27/476) (Table 3). The coincidence rate of B. caballi between the test card and cELISA was 97.90% (466/476) (Table 2). The results indicated that our newly developed test card was able to detect T. equi and B. caballi infections. The testing of the 476 samples showed that the test card correctly identified T. equi at rates of 86.15% (56/65) and 98.05% (403/411); similarly, the test card enabled the detection of B. caballi samples at rates of 85.19% (23/27) and 98.66% (443/449) (Table 2). Therefore, our newly developed test card agreed well with the OIE-recommended cELISA method, which can be used in clinical testing. The results also indicated that the newly developed card can test serum samples from not only horses but also donkeys. From the test results of these samples, we concluded that the positivity rate of T. equi was higher than that of B. caballi. Samples in most of the provinces were found to be positive for T. equi, but only samples from Xinjiang, Inner Mongolia, Heilongjiang, and Shaanxi were found to be positive for B. caballi. Among the 15 provinces, Tibet had the highest infection rate for T. equi, with a positive rate of 40%, and Xinjiang had the highest positive rate of B. caballi, reaching 50%.

**TABLE 1:** Comparison of the test card with the cELISA method for the detection of T. equi in clinical samples

Sample source	No. of samples	No. of samples with result using:			
		Test card		VMRD cELISA	
		Positive	Negative	Positive	Negative
Beijing	24	1	23	0	24
Guizhou	24	3	21	3	21
Hebei	24	2	22	6	18
Heilongjiang	30	12	18	12	18
Inner Mongolia	154	29	125	26	128
Liaoning	24	0	24	0	24
Ningxia	20	0	20	0	20
Qinghai	24	0	24	0	24
Shaanxi	20	5	15	5	15
Shanxi	24	0	24	1	23
Sichuan	24	0	24	0	24
Tibet	24	10	14	9	15
Wuhan	24	0	24	0	24
Xinjiang	16	2	14	3	13
Yunnan	20	0	20	0	20
Column totals	476	64	412	65	411

**TABLE 2:** Coincidence rates of the test card and cELISA

Test card result	No. (%) of samples with result using VMRD cELISA					
	*T. equi*			*B. caballi*		
	Positive	Negative	Total	Positive	Negative	Total
Positive	56 (11.76)	8 (1.68)	64 (13.45)	23 (4.83)	6 (1.26)	29 (6.09)
Negative	9 (1.89)	403 (84.66)	412 (86.55)	4 (0.84)	443 (93.07)	447 (93.91)

Total	65 (13.66)	411 (86.34)	476 (100)	27 (5.67)	449 (94.33)	476 (100)

**TABLE 3:** Comparison of the test card with the cELISA method for the detection of *B. caballi* in clinical samples

Sample source	Total no. of samples	No. of samples with result using:			
		Test card		VMRD cELISA	
		Positive	Negative	Positive	Negative
Beijing	24	0	24	0	24
Guizhou	24	0	24	0	24
Hebei	24	0	24	0	24
Heilongjiang	30	10	20	10	20
Inner Mongolia	154	8	146	2	152
Liaoning	24	0	24	1	23
Ningxia	20	0	20	0	20
Qinghai	24	0	24	0	24
Shaanxi	20	3	17	3	17
Shanxi	24	0	24	0	24
Sichuan	24	0	24	0	24
Tibet	24	0	24	0	24
Wuhan	24	0	24	0	24
Xinjiang	16	8	8	11	5
Yunnan	20	0	20	0	20
Total	476	29	447	27	449

## DISCUSSION

The clinical diagnosis of T. equi and B. caballi infection is typically achieved by performing blood smear staining to observe the parasite in erythroblasts; however, in the latency period of infection or in the case of chronic infection, thick blood smears for large-quantity sample tests are fundamentally difficult and less sensitive (29). Regardless of the fact that the CFT was previously the official regulatory test for establishing piroplasmosis status before travel to a non-endemic country, low sensitivity limited its wider application as the diagnostic test of choice for chronic infection (30, 31). In addition, IFA is a common detection method (32), but this method is time-consuming and requires technical expertise. ELISA has been used to detect antibodies against T. equi and B. caballi, but due to the need for special equipment, it is time-consuming and not suitable for use in on-site detection (33, 34). As horses and donkeys in China are mostly raised in fields, these methods cannot meet the needs of field testing. Complex farm environments are not suitable for conducting precise experimental operations. Most farms do not have qualified laboratory testing equipment, let alone professional laboratory operators, so the demand for simple and convenient testing methods is high.

The use of GICG strips is becoming increasingly popular and has many advantages, including low cost, rapidity, ease of performance, and interpretation of results. The whole test process takes only approximately 10 to 15 min, including sample treatment time, and the detection results are directly visible to the naked eye, with no requirement for expensive instruments and sophisticated laboratory equipment. The coexistence of the two EP pathogens (T. equi and B. caballi) in equids makes it very complicated to effectively prevent and control the disease. The two pathogens cause similar symptoms but are treated differently. Therefore, the two pathogens need to be distinguished. Current serological diagnosis methods are all tested separately, requiring two tests on the sample. In this study, based on the recombinant antigen and the prepared MAbs, we linked the detection strips for T. equi and B. caballi together in a test card so that one card in one detection can test for both diseases and can distinguish between T. equi and B. caballi infection. In field testing, the method can allow the operator to avoid carrying too many detection items, which is more convenient and helpful for observing the results and reduces operator error.

The results indicated that the test card has good specificity and high sensitivity. We tested serum samples from 15 provinces in China by applying the newly developed test card. The test card can also test serum samples from donkeys. The results showed a high rate of coincidence with the results using the VMRD company’s kit. At the same time, from the analysis of the tested samples, the positive rate of T. equi (13.66%) was higher than that of B. caballi (5.67%). From the geographical distribution analysis, the prevalence of EP in the north is higher than that in the south in China. Previous studies (35) have shown that the seroprevalence of T. equi ranges from 1.0% in Shanxi Province to 37.6% in Yunnan Province, and the seroprevalence of B. caballi ranges from 3.2% in Guangdong Province to 77.6% in Ningxia Province. However, these studies focused on local areas and had limited sample sizes and therefore could not represent the seroprevalence of EP across the whole country. As shown in a review of the global distribution of EP (36), the worldwide seroprevalences of T. equi and B. caballi were 33.2% (95% confidence interval [CI], 32.69 to 33.65) and 20.5% (95% CI, 19.98 to 20.93), respectively. Our results indicate that the seroprevalences of T. equi and B. caballi in China are lower than those worldwide.

The test card developed in this study should be useful for clinically diagnosing T. equi and B. caballi. A rapid, simple, and specific method targeting EMA1 and BC48 for T. equi and B. caballi antibody detection in equines was developed and optimized. EMA1 is the merozoite antigen of T. equi, and BC48 is the rhoptry protein of merozoites of B. caballi; good immunogenicity is relatively conserved, so the antigen is a good diagnostic antigen. Our results indicated that this immunochromatographic card assay provides a fast, reliable, and portable prediagnostic tool for T. equi and B. caballi, facilitating the preinspection process and subsequent confirmation and control of infected equids.

## MATERIALS AND METHODS

### Ethical approval.

This study was performed in strict accordance with the recommendations in the Guide for the Care and Use of Laboratory Animals of the Ministry of Science and Technology of the People’s Republic of China. The protocols were approved by the Committee on the Ethics of Animal Experiments of the Harbin Veterinary Research Institute (HVRI) of the Chinese Academy of Agricultural Sciences (CAAS).

### Serum samples.

In 2020, 476 clinical serum samples were collected from horses and donkeys in 15 provinces of China. The serum samples positive for T. equi, B. caballi, E. coli, Salmonella *abortus equi*, S. equi, EHV-4, EAV, B. mallei, EIAV, and T. evansi and the serum samples negative for T. equi and B. caballi were all stored in our laboratory.

### Preparation of recombinant proteins EMA1 and BC48.

Total DNA was extracted from blood samples positive for T. equi and B. caballi using the whole-blood DNA extraction kit (Tiangen, China). Based on the EMA1 gene sequence (GenBank accession number AB015214.1), PCR primers spanning an 825-bp amplification product (sense, 5′-ATGATTTCCAAATCCTTTGC-3′; antisense, 5′-TTAGTAAAATAGAGTGGAGAATGC-3′) were designed, with the inclusion of BamHI and NotI restriction sites, respectively. The amplified fragment was cloned into the expression vector pET-30a (Novagen, Germany) to construct the recombinant plasmid pET-30a-EMA1. The BC48 gene (GenBank accession number AB017700.1) was amplified from the genomic DNA of B. caballi-positive blood samples using the primer pair Bc48-F/Bc48-R (sense, 5′-ATGGCTCCCAGCGACTCTG-3′; antisense, 5′-CTATTTCTCCAATAAATTATCGGCC-3′), with the inclusion of BamHI and NotI restriction sites, and was ligated into the prokaryotic expression vector pET-32a (Novagen, Germany) to construct the recombinant plasmid pET-32a-BC48.

After being identified by restriction analysis and sequencing, the right fusion expression plasmid and the control vector were introduced into E. coli BL21 cells (Tsingke, China). The transformants were cultured in Luria Bertani (LB) broth supplemented with ampicillin or kanamycin (50 μg/mL) in a shaking incubator (180 rpm) at 37°C for approximately 3 h. When the OD_600_ reached 0.6 to 0.8, IPTG was added to the medium to achieve a final concentration of 1 mM to induce protein expression. The samples were further cultured for 6 to 8 h, after which the cultures were centrifuged at 8,000 × *g* at 4°C for 10 min. Cells were harvested and lysed by brief sonication in phosphate-buffered saline (PBS) (0.1 M, pH 7.4). The bacterial suspension was sonicated for 15 min to obtain a clear lysate. The EMA1 and BC48 proteins were purified by affinity chromatography using a Ni-nitrilotriacetic acid (NTA) agarose column system (Genscript, China) according to the manufacturer’s protocol. The recombinant EMA1 protein and BC48 protein were analyzed by SDS-PAGE. The correctly identified EMA1 and BC48 proteins were stored at −80°C.

### Generation of monoclonal antibodies against EMA1 and BC48.

The purified EMA1 and BC48 proteins were emulsified with equal volumes of Freund’s complete adjuvant (Sigma, USA) and used to immunize specific-pathogen-free 6-week-old female BALB/c mice (purchased from Liaoning Changsheng Biotechnology Co., Ltd., China) at approximately 100 μg per mouse. At 2-week intervals, two 100-μg boosters of purified antigen mixed with Freund’s incomplete adjuvant (Sigma, USA) were administered. The titers of EMA1 antibody or BC48 antibody in serum samples of immunized mice were monitored by indirect ELISA with EMA1 or BC48 as the antigen. Mice with the highest antibody titers were inoculated intraperitoneally with 100 μg of EMA1 or BC48. Then, the immunized mice were subjected to hybridoma cell preparation 5 days after the final booster. Spleen cells were harvested from immunized BALB/c mice and fused with SP2/0 cells during the logarithmic phase in the presence of PEG 1450 (Sigma, USA). The resulting hybridoma cells were cultured in RPMI 1640 medium (Sigma, USA) that contained HAT (hypoxanthine-aminopterin-thymidine) (Sigma, USA) and HT (hypoxanthine-thymidine) (Sigma, USA) for 10 to 14 days.

The hybridoma-secreting MAbs were selected by indirect ELISA. The hybridoma cells that tested positive by indirect ELISA were used for further subcloning. The antibody titers of the EMA1- or BC48-immunized mice and the supernatant of the hybridoma cell culture and the MAb titer were determined or examined by indirect ELISA with EMA1 or BC48 as the coating antigen. Briefly, each well of a 96-well microtiter plate was coated with a 100-μL aliquot of the EMA1 or BC48 protein (1 ng/μL in PBS), and the plate subsequently incubated overnight at 4°C. The plate was then washed 3 times with PBST (0.05% Tween 20 in PBS) and incubated with mouse sera or MAb for 2 h at 37°C. After another washing, the plate was incubated with 100 μL of horseradish peroxidase (HRP)-conjugated goat anti-mouse IgG (KPL Company, Gaithersburg, MD) (1:5,000) for an additional hour at 37°C. The plate was then washed again, and the colorimetric reaction was developed using TMB (3,3′,5,5′-tetramethylbenzidine) (Taitianhe Biotechnology Co., Ltd., China) for 10 min at 25°C. The reaction was stopped with 2 mol/L H_2_SO_4_, and the OD was read at 450 nm. An OD greater than 0.400 was considered positive. The monoclonal cell lines were expanded and cultured, and the cell supernatants were obtained to identify the antibody subtypes according to SBA clonotyping system-HRP (Southern Biotech, USA) instructions.

Ascites fluid containing MAb was prepared by injecting 5 × 10^5^ selected hybridoma cells into the abdominal cavity of each mouse. One week prior to the ascitic fluid culture, mice were injected with Freund’s incomplete adjuvant (Sigma, USA). The ascitic fluid containing the MAb was purified using a protein G perfusion affinity chromatographic column (GE Healthcare, USA). The purified MAbs were dialyzed in PBS (10 mmol/L) and determined using an UV spectrophotometer at 280 nm.

### Preparation of colloidal gold.

Briefly, 2 mL 1% chloroauric acid solution was added to 198 mL ultrapure water to make a 0.01% chloroauric acid solution. In a conical bottle, a 0.01% chloroauric acid solution was heated to the boiling point, which was followed by the addition of 3.2 mL of a 1% trisodium citrate solution under continuous gentle stirring. The reaction mixture was boiled until the color turned burgundy to the naked eye and was transparent with no precipitate or floating particles; this process took approximately 20 min. The colloidal gold solution was stored in a dark bottle at 4°C. The quality of colloidal gold particles was examined by transmission electron microscopy (TEM) (H7650; Hitachi, Japan) (37).

### Preparation of colloidal gold conjugate.

The optimum pH for labeling colloidal gold with EMA1 or BC48 was 4, which was determined by varying the amount of 0.2 M K_2_CO_3_. Amounts of 200 mL of colloidal gold were mixed with 4.8 mL of EMA1 (4 mg/mL) or BC48 (4 mg/mL) and incubated at room temperature for 1 h. A 10% bovine serum albumin solution was added under electromagnetic stirring until the final concentration was 1%, and the mixture stirred continuously for 60 min. The colloidal gold-labeled protein EMA1 or BC48 was placed at 2 to 8°C and centrifuged at 2,000 rpm for 20 min, and the supernatant was collected. The supernatant was maintained at 2 to 8°C and centrifuged at 10,000 rpm for 20 min. The supernatant was discarded, and the precipitate was dissolved in 6.6 mL colloidal gold standard diluent and stored at 2 to 8°C away from light. The colloidal gold standard diluent was prepared with 1.0 g bovine serum albumin, 5.0 g sucrose, 2.0 g trehalose, and 100 mL Tris(hydroxymethyl)methyl aminomethane (20 mmol/L, pH 8.6) buffer, stirring until completely dissolved, and then 150 μL Triton X-100 was added and the mixture stirred evenly and filtered using a 0.22-μm filter for sterilization.

### Preparation of the immunochromatographic strip.

An immunochromatographic strip that included a sample pad, a conjugate pad, a nitrocellulose membrane, and an absorbent pad was prepared as shown in Fig. 1. Colloidal gold probe was added to the conjugate pad and dried at 37°C for 2 h. The bottom of the immobilized nitrocellulose membrane (NC membrane; Millipore, USA) was coated with MAb 1A12 (EMA1) (1.0 mg/mL) and EMA1 protein (0.4 mg/mL) to form the control line (C line) and test line (T line), respectively. The top of the immobilized NC membrane (Millipore, USA) was coated with MAb 1A3 (BC48) (1.0 mg/mL) and BC48 protein (0.4 mg/mL) to form the control line (C line) and the test line (T line), respectively. The NC membranes were dried for 12 h at 37°C. Finally, the sample pad, conjugate pad, fixed NC membrane, and absorbent pad were cut into 4-mm-wide strips, sequentially overlapped, and assembled into one test card.

### Specificity and sensitivity of the test card.

To evaluate the specificity of the card, serum samples positive for T. equi, B. caballi, E. coli, Salmonella *abortus equi*, S. equi, EHV-4, EAV, B. mallei, EIAV, and T. evansi and serum samples negative for T. equi and B. caballi were simultaneously tested at a dilution of 1:10. The card’s sensitivity was determined with a serial dilution of a seperate serum sample positive for T. equi or B. caballi, which was diluted at ratios of 1:2, 1:4, 1:8, 1:16,1:32,1:64,1:128,1:256, and 1:512 with PBS.

### Comparative analysis of the clinical samples.

The test card and the cELISA kit (VMRD, USA) were used in parallel to detect antibodies against T. equi and B. caballi. Serum samples were collected from 456 horses and 20 donkeys in 15 provinces across the country, as shown in Fig. 2. OIE considers cELISA to be the standard method. We used the VMRD cELISA kit in this study, which is widely used worldwide. Colloidal gold was used to drop 10 μL serum into the sample well, and then 90 μl serum diluent was added and the serum was maintained for 10 to 15 min to observe the results. We also performed ROC curve analysis as a statistical tool for the diagnostic evaluation of the GICG test card assay in surveillance samples, where the value for the AUC was employed to assess the accuracy of GICG test card assays (an AUC of 1 indicates perfect discriminatory value; an AUC of 0.5 or less indicates no discriminatory value).
